# RuleBender: integrated modeling, simulation and visualization for rule-based intracellular biochemistry

**DOI:** 10.1186/1471-2105-13-S8-S3

**Published:** 2012-05-18

**Authors:** Adam M Smith, Wen Xu, Yao Sun, James R Faeder, G Elisabeta Marai

**Affiliations:** 1 Department of Computer Science, University of Pittsburgh, Pittsburgh, 15260, USA; 2 Department of Computational and Systems Biology, University of Pittsburgh, Pittsburgh, 15260, USA

## Abstract

**Background:**

Rule-based modeling (RBM) is a powerful and increasingly popular approach to modeling cell signaling networks. However, novel visual tools are needed in order to make RBM accessible to a broad range of users, to make specification of models less error prone, and to improve workflows.

**Results:**

We introduce RuleBender, a novel visualization system for the integrated visualization, modeling and simulation of rule-based intracellular biochemistry. We present the user requirements, visual paradigms, algorithms and design decisions behind RuleBender, with emphasis on visual global/local model exploration and integrated execution of simulations. The support of RBM creation, debugging, and interactive visualization expedites the RBM learning process and reduces model construction time; while built-in model simulation and results with multiple linked views streamline the execution and analysis of newly created models and generated networks.

**Conclusion:**

RuleBender has been adopted as both an educational and a research tool and is available as a free open source tool at http://www.rulebender.org. A development cycle that includes close interaction with expert users allows RuleBender to better serve the needs of the systems biology community.

## Introduction

Systems Biology researchers study the mechanisms and effects of intracellular chemical interactions. Molecules in an organism act as catalysts for long chains of reactions that lead to an observable response such as gene expression or production of a protein. The field of study that focuses on paths along these reaction networks is known as *cell signaling*. Better understanding of cell signaling can lead to advances in drug discovery and the treatment of diseases like cancer, Parkinson's, and Alzheimer's.

Traditional studies of cell signaling involve chemical experimentation wherein the researchers measure the concentrations of molecules throughout the course of a reaction via microscopy or biochemical methods. This molecular concentration data from laboratory experiments can also be used to construct ordinary differential equations that represent the cell signaling network over the time course of a series of reactions. Such mathematical models can then be simulated in order to make predictions that the data alone cannot generate.

Rule-based modeling (RBM) allows for the construction of an executable model that contains a starting set of molecules with possible interaction behaviors. These models are then simulated in order to produce a complete reaction network. If the network matches known cell signaling data, then the model is assumed to be correct and can be used to construct hypotheses about the biological system in question. Thanks to the relatively low cost of model alteration and simulation compared to laboratory experimentation, the RBM approach can be used to gain insight about a reaction network, and can help speed up the discovery of new drugs and therapies.

While the potential benefits of RBM to biology are outstanding, the process of building an RBM from experimental data and detecting and correcting modeling errors (i.e., debugging) can be tedious and frustrating. RBMs are typically defined by the user via a text file. The user defines a set of molecules and proceeds to write rules governing their interaction that are derived from specific biomedical literature knowledge of the biological system. Although individual rules are easy to write, it is often difficult to fully grasp the implications of a set of rules. The challenge in grasping the global perspective is particularly acute when trying to understand models written by different researchers. This problem complicates debugging and reduces the accessibility of RBM, especially for users with limited programming experience. We hypothesize that visual global/local model exploration can help with these tasks. Beyond modeling difficulties, simulating and analyzing RBMs pose additional challenges.

The goal of this collaborative project was to facilitate RBM construction, simulation, and analysis in an integrated system. Given the combination of spatial and abstract information typical to RBM, and the challenges briefly outlined above, we pursue a visual backbone for such a system. Our first contribution is a description of the typical RBM workflow, followed by an analysis of the tasks and potential sources of error in model construction and analysis. This information was collected through close interaction with systems biologists. Secondly, we propose a set of complementary visual encodings and visualization strategies to be used during the model construction and analysis process. Our third contribution is the implementation and description of the discussed features in the open source system RuleBender. Next, we evaluate this system on two case studies and report feedback both from expert users and from classroom usage. Finally, we contribute a discussion of the design decisions behind the system and of the lessons learned through our collaboration with biology researchers.

## Background

### Computational complexity of molecular processes

Bioinformatics researchers are concerned with discovering the structure and interactions of molecules, DNA, and proteins. In this paper we refer to all major structures analyzed by researchers as *molecules*. Each molecule is composed of specific substructures that are called *domains*. The interactions between molecules are caused in fact by interactions among the domains of those molecules.

Cell-signaling systems involve an intricate network of protein-protein interactions. These interactions can have a number of consequences, including the post-translational modification of proteins, the formation of heterogeneous protein complexes in which enzymes and substrates are co-localized, and the targeted degradation of proteins. For understanding the system dynamics, the details that are most relevant are typically found at the level of protein sites or domains that are responsible for protein-protein interactions. Despite the high relevance of the site-specific details of protein-protein interactions for understanding system behavior, models incorporating these details are uncommon. Models that incorporate protein-site details are generally difficult or impossible to specify and analyze using conventional methods, largely because of the combinatorial number of protein modifications and protein complexes that can be generated through protein-protein interactions (i.e., *combinatorial complexity*) [1].

### Rule-based modeling of molecular processes

The limitations of conventional approaches to model specification have prompted the development of formal languages specially designed for representing proteins and protein-protein interactions [2]. BioNetGen is a language and software framework that uses graphs to represent protein-protein interactions [3]. BioNetGen allows site-specific details and dynamics of protein-protein interactions in a systematic fashion. New algorithms permit efficient simulation of rule-based networks of virtually any size and complexity [4,5].

A BioNetGen input file contains definitions of *molecules*, *reaction rules*, chemical and mathematical constants, initial molecule populations, and simulation instructions. The models include definitions for the molecule itself, and also its domains and any associated bonds. Domains may also have associated *states*, e.g. phosphorylated or unphosphorylated. Each rule is defined by a set of reactants that are composed of molecules, domains, and states; followed by the post-reaction product which may include new bonds, broken bonds, or changed states of domains. In these rules, the molecules, domains with states, and bonds that are required for the reaction but are not changed by it are called the *reaction context*. Conversely items that are changed by the reaction are termed the *reaction center*.

In BioNetGen rules are applied iteratively to species to generate the partial or full set of reachable species and reactions. The resulting reaction network, composed of these species and reactions, is then simulated to obtain the population of each species as a function of time using for example numerical integration of ODE's or stochastic simulation methods. An alternative approach is the so-called network-free method that performs a discrete event simulation on an instantiated set of molecules [4].

## Related work

Graphical representations of molecular processes -- primarily state-transition diagrams -- have been in use in biology textbooks as early as 1949 [6], and later on transitioned in the same diagram form into database systems such as KEGG, EMP, and EcoCyc [7-9]. Software systems for pathway design such as NetBuilder, Patika, JDesigner, or CellDesigner [10-13] have introduced additional notations for the same basic graph structure, while with the development of genomics new notations -- such as arcs, edges, and glyphs -- have been proposed for signaling pathways, and for incomplete or indirect information [14,15].

Kohn added a formal syntax to the set of symbols above that describes interactions and relationships of molecules in a rigidly defined schema known as Molecular Interaction Maps (MIM's) [16]; MIM's provide guidelines and approaches to drawing static, schematic representations of signaling pathways. Kohn's MIM notation was followed by additional proposals [17,18] describing process diagrams with both standard symbols and defined grammars. In a recent effort, the Systems Biology Graphical Notation (SBGN) proposal [19] is attempting to establish a community standard for biological notation.

The important observation here is that, while many graphical representations of molecular processes have been proposed, the construction of these representations is not automated, and the diagrammatic representations themselves are either non-computable or have limited computability due to combinatorial complexity. In other words, novel software tools are needed that can convert a graphically represented model into mathematical formulas for analysis and simulation.

A large number of systems have been developed to facilitate pathway construction and analysis, most notable among them GenMAPP [20], Cytoscape [21] and its recent extensions [22], PathwayAssist [23], Patika [11], GScope [24], GeneShelf [25] and GeneSpring [26]. For an extensive review of many of these systems, see Saraiya et al. [27]. While many of these systems have complementary strengths in terms of the user requirements identified by Saraiya et al. [27], such as collaboration, context overlay, assistance for pathway construction, highlighting temporal information, etc., they are generally designed to facilitate integration of experimental data into the analysis process, with no emphasis on computational simulation. Recent commercial attempts at combining visualization with simulation and modeling [28] have employed rule-based languages, although the resulting visual representations are minimalistic and, to the best of our knowledge, not formally specified.

Novel techniques are needed to integrate modeling, computational simulation, and visual analysis of biochemical systems in order to construct models of signaling pathways that are accurate, visually understandable, computable, and multi-scale.

## Workflow and task analysis

Our first contribution is an analysis of the typical RBM workflow; of the tasks associated with this type of modeling, simulation and analysis; and finally an analysis of the potential modeling error sources. These analyses are based on on-site interviews conducted with RBM researchers.

The typical RBM workflow starts when a modeler is assigned a particular biological system and is asked to investigate certain properties of the system (e.g., the effect of different parameters on the model output; or finding what assumptions about the model are critical). The modeler begins by performing a literature search for the model; the required inputs are a set of molecules, their interactions, and parameters that quantify the concentration and strength of the interactions (in the form of rate constants). Biological databases have considerable information about biomolecules and their interactions but contain little information about parameters, which must be obtained from manual searching of the literature. The modeler then proceeds to write the system components and the set of rules describing the behavior of the system. Once a working model has been defined, an RBM can be simulated using a number of different approaches including ordinary differential equations, stochastic simulations, or particle-based stochastic simulations. The output must be then analyzed and compared against other results. The typical workflow relies on an external plain text editor, command console, and external plotting tools for displaying simulation results, which is inconvenient because it requires modelers to switch between different tools over repeated cycles of model editing and simulation. The process gets further complicated when exploring alternative simulations and models.

To design our system, we extracted first the list of eight most frequently performed RBM tasks shown in Table 1 (shown are also the scores attained by RuleBender). This set of tasks informed our system specification: at a minimum, the system needs to provide debugging capabilities, it needs to bridge model construction, simulation, and analysis, and needs to provide parameter scanning capabilities. Next, prototyping revealed the necessity for clear yet concise visual abstractions that scale well with the possible sizes of the data sets to be visualized. Finally, the interviews revealed additional system requirements such as an efficient workflow; a stand-alone system as opposed to a web-based one, on account of latency concerns; a system that is cross-platform and easy to install; and a tool that is usable with minimal training.

**Table 1:** RBM Tasks and RuleBender Scores

Index	Task	Score (1 to 5)
*T1*	Compose a model from scratch.	4.2
*T2*	Find and correct an error in a model.	4.8
*T3*	Understand relationships between rules in the model - do they have overlapping reactants, products, etc.?	4.4
*T4*	Modify an existing model and run simulations to compare results with those of the original.	4.2
*T5*	Generate a network; examine species and reactions.	4.4
*T6*	Run a parameter scan. Examine overall results and look at results for individual trajectories.	4.8
*T7*	Compare results of scanning a parameter in two different models.	4.4
*T8*	Find a set of parameters that makes the model behave in a specific way.	3.4

In attempting to provide debugging capabilities for such a system, we next discussed potential modeling pitfalls with our systems biology collaborators. Three types of errors became apparent: syntactic, semantic, and biological errors. Syntax errors are typos or incorrect usage of the modeling language. These syntax errors are the easiest to detect and repair, by using an appropriate editor with syntax checking, syntax highlighting and valid parameter name recognition. The second class of errors, semantic errors, occurs when a modeler produces code that is syntactically correct but is not the intended structure regardless of whether the intended model is biologically correct. For example, the model syntax is correct, but one rule introduces an unwanted complex; multiple rules interact, creating an unwanted effect; or the modeler simply misunderstood the model syntax/semantics. According to our end users, almost all interesting errors were of this second, semantic type. Finally, biological errors occur when a user misinterprets the literature and aims to create a model that is incorrect with respect to known network structure; alternatively, the user may create a correct model but does not include the correct initial concentrations or reactions rates. Due to the size and complexity of some models it may be impossible to detect such biological based errors without expert knowledge. However, the difficulties of detecting semantic and biological errors can be alleviated with visual representations of the model that focus on the molecule structure and interactions.

## RuleBender

To address the current difficulties of model creation and repair, simulation, and analysis we pursue an integrated design that includes (i) an editing environment, (ii) built-in simulation execution, (iii) complementary visual representations of models, and (iv) simulation analysis capabilities in a multi-pane visual framework that collects the entire RBM workflow. Given the complementary nature of the information involved in RBM, our top design uses a linked multi-view approach. The views are organized according to the workflow we identified earlier.

**Figure 1:**
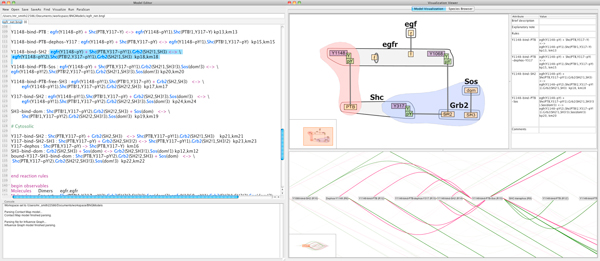
**The RuleBender interface**. Shown are the Model Editor pane including console for text output (left) and the Visualization Viewer pane (right). The Visualization Viewer shows two complementary visual encodings corresponding to the text model in the Editor: the interactive contact map (top), and part of the influence graph for this model (bottom). RuleBender's main features include syntax checking, syntax highlighting, visual global model exploration with linked views, integrated execution, support for multiple simulation modules, simulation journaling, interactive plotting including comparison of multiple datasets, and parameter scanning.

The visual interface incorporates text editing, visualization, and simulation execution in order to facilitate a faster and more productive RBM workflow. Three main vertical panes are used. The first pane (Figure 1) provides a text-based Model Editor and a console window. In addition to standard text editor capabilities, the Model Editor provides a number of useful features for creating and editing RBMs in BioNetGen Language (BNGL) format.

The second main pane, the Visualization Viewer, is reserved for global and local visual representations of the RBM; its purpose is to assist the modeler in the process of debugging the RBM. These interactive visual representations help modelers form complex model structures and internal interactions progressively, rather than trying to build and keep track of a complete mental model from the start. The visual representations are generated automatically from the text-based representation (as later described), and updates in the Model Editor are reflected in the Visualization Viewer. Logic errors in the RBM that cause parsing errors in the Visualization Viewer are reported in the console window of Model Editor (Figure 1). The human closes the loop, by repairing in the Model Editor the errors reported in the console, as well as any semantic errors detected via visual analysis.

After the first iteration of model construction, the modeler can generate an explicit network of the modeled system, and then run multiple simulations based on the generated network. The Model Editor provides integrated execution of BioNetGen simulator commands through menus and toolbar buttons; these actions include parameter scanning operations that allow the interactive study of the effects of varying the value of a single model parameter. At this point, the Visualization Viewer pane is replaced by the third pane, the Simulation Results Viewer. The two Viewers can also be laid side-by-side. Based on the analysis, the modeler could start a new iteration of modeling and simulation in order to revise the model or explore the effects of small model changes.

Below we detail the data abstractions and algorithms specific to the Model Editor, Visualization Viewer, and the Simulation Results Viewer. Design decisions and revisions of these abstractions and algorithms were made in close collaboration with our expert end-users.

### Model Editor

The Model Editor window provides an environment for creating and editing RBMs in text-mode - the traditional approach to specifying biochemistry rule-based systems. The window is composed of a fully featured text editor and a console for reporting model syntax errors and simulation logs to the user. To facilitate comparative model exploration the Model Editor supports simultaneous editing of files through tabbing.

In order to expedite model construction, the Model Editor includes a BNGL model template for creating new files. Following the current specification of the BNGL language, each BNGL file must define a text block for parameters, molecule types, seed species, reaction rules, observables, and simulation actions. The *parameters block *holds numerical constants or equations that define concentrations of chemicals or rates of reaction rule occurrence. The *molecule types block *allows the user to declare the basic molecules that will appear in the model. In contrast, the *seed species block *states the starting collection of molecules for simulation and network generation. The *reaction rules block *is a collection of all of the possible chemical behaviors of the system. The *observables block *provides the user with the ability to mark certain molecules or collections of molecules for observation in the results of a simulation. Finally, the *simulation actions block *comprises a list of instructions for how to execute a model. BNGL simulation instructions support generating and simulating a network, managing molecule concentrations and parameter values, and saving models. Code folding hides details of completed text blocks so that the unfinished features become more visually salient. Syntax highlighting of keywords and language features - one of the earliest user-requests - also assists with understanding and debugging the syntax of the model. Text selection results in an automatic search for the selected text and all found occurrences are highlighted.

In the process of incremental model construction modelers make syntax errors that are easily detected by a parser and reported through the console. However, semantic and biological errors are difficult to detect based on the textual representation only. To further support model exploration and debugging the Visualization Viewer provides both global and local views of the model currently loaded in the Model Editor: interactive contact maps and influence graphs.

### Interactive Contact Map

**Figure 2:**
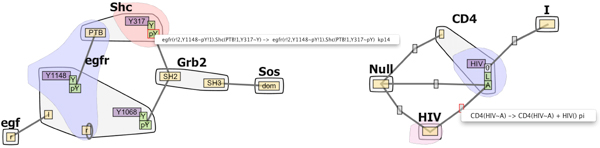
**The Contact Map**. Contact Maps without (left) and with (right) hub nodes. Molecules are represented as larger nodes (light gray) while domains and domain states (yellow, orange and purple) are represented as smaller sub-nodes in the molecules. State nodes (green and dark gray) are adjacent to the domain sites to which they apply. Reaction rules are mapped to edges (rules that indicate the creation or destruction of a bond between these two domains) and state nodes (rules that indicate state changes). Selecting a state-node (red boundary on the left) lists all rules that indicate that state change. Similarly, selecting an edge (not shown) lists all rules that create or destroy bonds between the linked domains. Selecting one rule from such a list marks the reaction context in blue and the reaction center in pink. Hub nodes are associated with rules that define molecular level interactions without domains involved, such as the degradation of proteins. Selecting a hub node lists all rules involving the linked molecules as shown on the right.

The first visual encoding we propose is the Interactive Contact Map (Figure 2), a concise, scalable representation that provides a global view of the RBM. This encoding is an interactive graph representation of the molecules and the reaction rules governing the system. Recall that in RBM, molecules are described as structured objects that are composed of domains that can have states and can bind to each other, both within a molecule and between molecules. Also, reaction rules are the generators of species and reactions, which define all the interactions. Given that reaction rules are an essential part of the model, the Contact Map needs to show not only the involved molecules, but also an overview and details of the various reaction rules.

#### Data abstraction and representation

To keep the Contact Map concise and scalable, the molecules and internal domains defined in the model are displayed only once in the graph. Molecules are rendered as large gray nodes and domain sites are smaller internal nodes. domain states (such as unphosphorylated Y and phosphorylated pY), may be specifically required in certain reaction rules, and so are also displayed as nodes cascading from the domain sites to which they apply (Figure 2).

To add rule information to this representation, we next analyze the various reaction rules and find they fall into three categories. The most common and simple type of reaction rule defines bond creation or destruction between domains. A bond can only exist between two domains. For this type of rule, an edge connecting two domain nodes is created in the Contact Map. Reaction rules that involve the same bond will be mapped to the same edge in the graph. In certain rules, specific domain states may be required in order to create or destroy the bond. In those cases the state node instead of the domain node is connected by an edge.

The second type of reaction rule defines state changes of domains. A domain can only have one state at a time, and the state can be changed based on reaction rules. Adding an edge between two state nodes is not a good solution, because mapping two types of rules in the same way would cause confusion and adding more edges will increase clutter since the state nodes of one domain are positioned very close to each other in the graph. Given these limitations and the importance of the state information, this type of rule is mapped to the target state node via color: domains that have their states changed via a rule are shown in purple as shown in Figure 2. The last type of rule defines molecular level interactions without domains involved, such as the degradation of proteins. In this situation, a hub node and several edges will be created to connect each reactant and product molecule in the rule (Figure 2 right).

Next, we note that each rule has its own reaction center (the domains being modified by the rule) and reaction context (the domains are required for the rule to be applied but are not being modified). We use Bubble Sets [29] to display this information. The bubble sets algorithm draws an isocontour around all of the items in a particular set in order to more easily see set membership (light blue and pink in Figure 2).

**Figure 3:**
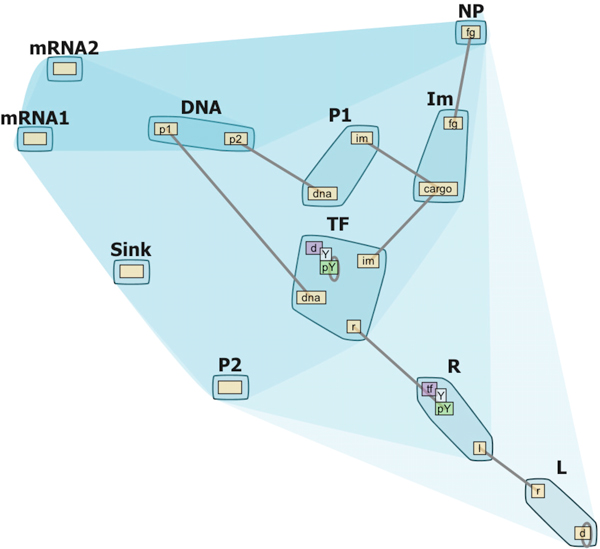
**Compartmental Contact Map**. Contact Map with molecule compartment hierarchy (extracellular, cytoplasmic, nucleus etc). The saturation of the convex hull encompassing a compartment indicates the hierarchical structure of the compartments; the outermost compartment is colored the lightest blue. All the members of a compartment can be moved as a whole unit to get a clear view of the hierarchical structure.

Finally, feedback from more recent end-users revealed the need for a visual representation of the various molecule compartments (extracellular, cytoplasmic etc.) shown in Figure 3. The compartmental localization of model domains can be displayed when this information is provided by the modeler.

#### Layout

We use force-directed layout algorithms [30] to draw the Contact Map in an aesthetically pleasing way while minimizing edge crossings. A small overview window of the Contact Map helps the modelers to navigate large graphs.

The different types of nodes were assigned colors using ColorBrewer [31], which in turn follows Tufte's principles for information encoding [32]. The primary nodes are shown in yellow (no state information), orange (state information but no state change), or purple (state change). Domain states are shown in green (state node with state change), or gray (state node without state change) (Figure 2 left).

Following the basic Visual Information Seeking Mantra [33], the Contact Map first gives an overview of the model. Pop-up menus provide filtering options such as showing or hiding state nodes in which case the endpoints of edges switch between domain node or state node accordingly. Details of molecules and reaction rules are shown on demand. Selecting an edge, a state node, or a hub node brings up a list of reaction rules, and selecting one of these rules brings up the bubble sets overlay highlighting the reaction context in blue and the reaction center in pink. Selecting a molecule brings up a list of external links of available online resources in an annotation panel (Figure 1).

### Influence graph

**Figure 4:**
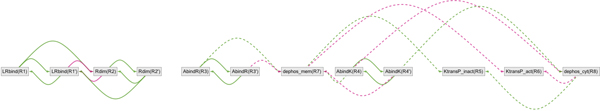
**The influence graph**. Nodes represent reaction rules while arcs represent influence between rules. Green/Magenta solid arcs represent fully activation/inhibition, and Green/Magenta dash arcs represent partial activation/inhibition. Filter options that show or hide activation/inhibition are provided through pop-up menus. Two separate groups of rule nodes (group1: the first four nodes, group2: the rest of the nodes). can indicate that the model is not complete.

While the Interactive Contact Map shows in a compact manner the connectivity between the molecules within a model, the relations among the reaction rules may provide further insight into the model behavior. An influence graph (Figure 4) is an abstraction of complex reaction networks; influence graphs were originally introduced for the analysis of gene expression in the setting of gene regulatory networks. We extend this concept to rule-based modeling. Rule-based influence graphs give an overall view of the activation/inhibition relation between the reaction rules that describe the behavior of a system.

#### Data abstraction and representation

We identify four types of relations between reaction rules: full activation, full inhibition, partial activation and partial inhibition. The difference between the full and partial is that full means the firing of the influencing rule will definitely affect the rate at which the second rule fires, whereas partial means the firing of the influencing rule may or may not affect the rate at which the second rule fires depending on which specific species or agents are transformed by the influencing rule.

**Figure 5:**
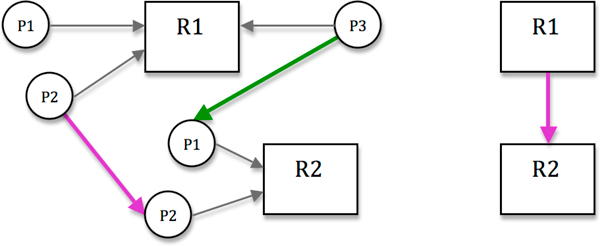
**Influence graph definition**. Prototype pattern relations (P) and rule relations (R) used to determine influence graphs: an intermediate graph (Left) is ultimately reduced to the simplified, final influence graph (Right). An arrow from P to R means that P is a reactant pattern of the R; for the reverse direction P is a product pattern of R. Green edges show activation relations and red ones show inhibition.

There are generally two steps to get the relation between two rules. The following description refers to the relation from Rule 1 to Rule 2. Recall that rules are composed of required reactants and post-reaction products. We use *patterns *to describe a component of the reactants or products that may overlap with another rule. Figure 5 shows an example of pattern relations and rule relations that can be used to construct an influence graph:

Step 1: Attempt to match all of the reactant patterns of Rule 2 onto the reactant patterns of Rule 1. If there is a full match, for example, from Pattern 2 of Rule 2 onto Pattern 2 of Rule 1 (as in Figure 5), then there is a full inhibition, as indicated by the red arrow in the left hand panel of Figure 5. A partial match indicates a partial inhibition. If there is no match of a reaction center element or conflict between any elements of the two patterns, then there is no inhibition. Similarly, pattern matching from product patterns of Rule 1 to reactant patterns of Rule 2 can be performed to obtain the activation information.

Step 2: With the relation information between the patterns of the 2 rules acquired in the previous step, we can summarize the information to get relations between the two rules. In the reduction a full influence should have higher priority than a partial influence.

Through iteration of the above two steps between all pairs of reaction rules within the model, the influence graph information is algorithmically constructed. Then we display the Influence Graph as a directed graph with nodes representing rules and edges representing relations between rules.

#### Layout

Similar to the Contact Map, we use colors, filtering, zoom in/out, focus plus context, and details on demand to design the visualization. Different colors [31] and shapes are applied to the edges to distinguish the types of relations: green was chosen for activation and magenta was chosen for inhibition. Dashed lines represent partial inhibition/activation and solid lines represent full inhibition/activation. Decorated edges were preferred to styled arrow heads to make the edge characteristics more easily visible at lower zoom levels. Activation and inhibition filtering operations are also provided. Selecting a rule node displays the rule text and filters the influence arcs related to this node (Figure 1).

We note that there are no certain patterns or obvious hierarchical structure among the relations. Therefore we chose a linear arc diagram design. All the nodes are arranged in a horizontal line, with nodes sorted according to their connectedness, and arcs connect nodes representing relations symmetrically. The length and height of an arc depends on the horizontal distance between two nodes. The direction of an arc becomes very clear in this layout. The arcs above the horizontal line point to the right while the arcs below the horizontal line point to the left. A small overview window of the Influence Graph is also provided in the Visualization Viewer to help the modelers to navigate large graphs.

Several graph-drawing approaches were attempted (and discarded after feedback) for rendering the influence graph - including circular layouts, force-directed layouts, and several variations of the linear display. Many of these attempts suffered from scalability problems. In the end, traits of the winning design were the linear, bilayered output (forward rules on the upper side, backward rules on the lower side), interactive filtering, providing the appropriate amount of detail (e.g., rule mnemonics as opposed to numbers), and the ability to link back to the textual representation.

### Simulation and simulation journaling

RuleBender provides flexible support for multiple simulation modules, including parameter scanning, and for simulation journaling. A Results Viewer and a Species Browser further allow interactive plotting of simulation results, including comparisons of multiple datasets, and visual exploration of the resulting species.

Simulation can be initiated after an RBM model has been constructed or loaded in the Text Editor. Certain RBM simulation techniques require that the full reaction network for the model be first generated. During network generation, the rules defined in the model are applied to the initial species until a user-defined maximum number of iterations is reached or until no new chemical species are produced. After a network has been generated, simulation of the network can be carried out by either numerical integration of ODE's or through a stochastic simulation of the model.

In contrast, on-the-fly simulation does not require a pre-existing network and generates the full network using the model rules as the simulation takes place. RuleBender supports network-free simulation through the NFSim package [34] which works entirely without network generation by using discrete-event particle-based techniques.

Simulation actions can be listed and executed in order. Intermediate versions of models that have been partially simulated or that are at equilibrium can also be saved for later use. Notably, RuleBender allows model-changing commands to be introduced in between these simulation actions. Examples of model-changing actions include altering the concentrations of species or setting new parameter values for the rates at which rules occur. For example, a network can be generated, simulated to equilibrium with a subset of its species, and then simulated again after introducing a predetermined concentration of another species.

RuleBender also supports a simulation technique called parameter scanning. In a parameter scan, the starting value of a single parameter is varied over many simulations in order to measure the effect of changing that parameter. Visually mining the relationships between parameter values and outcomes is a direction of future work.

To support simulation journaling, simulation run results are stored in individual directories. Each results directory is labeled with the model name and the time of the execution; the collection of results directories forms the *model simulation journal*. Each directory includes log files, a copy of the exact model and parameters that were executed, the generated network, and the results of the simulation. Time-series data resulting from simulations are stored in two files: CDAT files contain concentration data over time for all of the generated species individually, and GDAT files contain concentration data over time for the modeler-defined observables. An additional NET file contains supplemental information about the fully generated network.

### Simulation results and species browser

#### Results viewer

**Figure 6:**
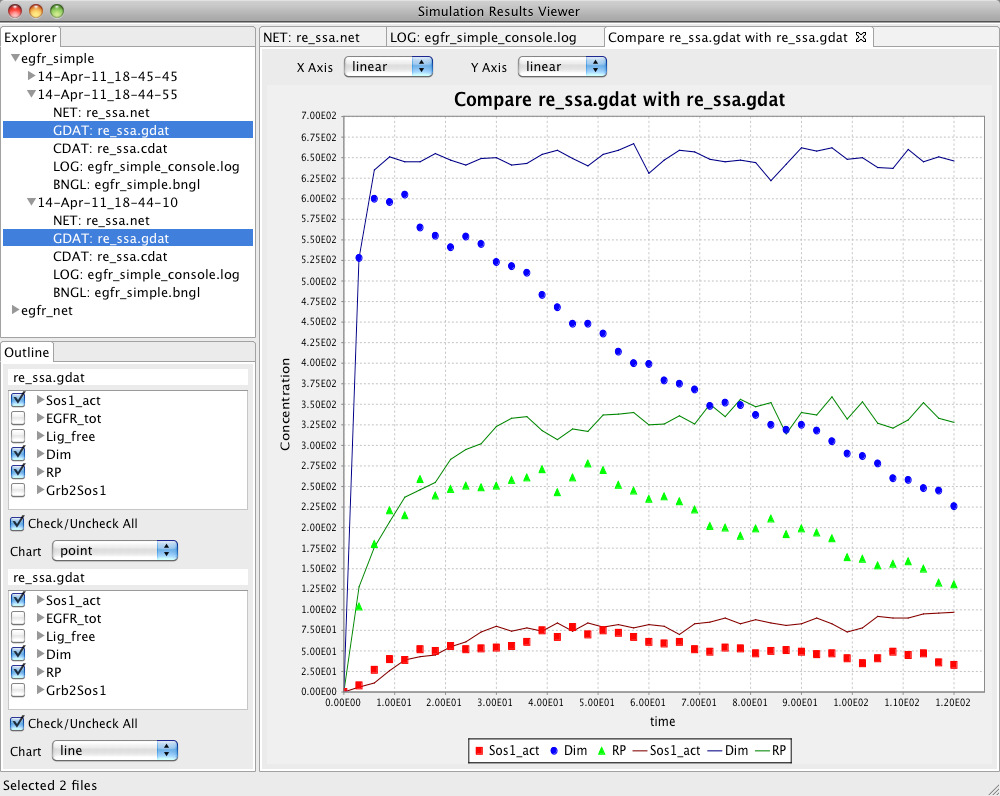
**The simulation results viewer**. The upper left quadrant of the window contains a file explorer for easy retrieval of the exact version of a particular model associated with a specific set of results; the bottom left quadrant shows the list of species or observables. Charts in linear or log scale show the time series for concentrations of chemical species and observables. Any number of species and observables can be compared in the same chart. Furthermore, multiple simulation runs can be compared in order to analyze the effects of changing the model. The example in the snapshot compares the results of two simulations (points and lines) with three observables selected individually.

The Simulation Results Viewer (Figure 6) provides support for exploring simulation journals, for interactive plotting including comparisons of multiple datasets, and for visual exploration of the resulting species.

The upper left pane of the of the Simulation Results Viewer contains a tree-based structure corresponding to the journal of the simulation results. Each node in the first level of the tree represents a single run of a simulation. When the user selects either of the simulation result files, the data are displayed in an interactive line or point chart in the large right pane. The modeler can analyze the results of multiple simulation runs using text, charts, and graphs. Following the end-user requirements, the charts support both line and point representations of the data and can be rendered with linear or log scale on both axes. Mouse brushing is used in order to zoom in and out on the chart. Below the results file tree viewer, the list of generated species or observables is displayed with a check box next to each element. Only the selected elements are shown in the chart.

#### Species browser

**Figure 7:**
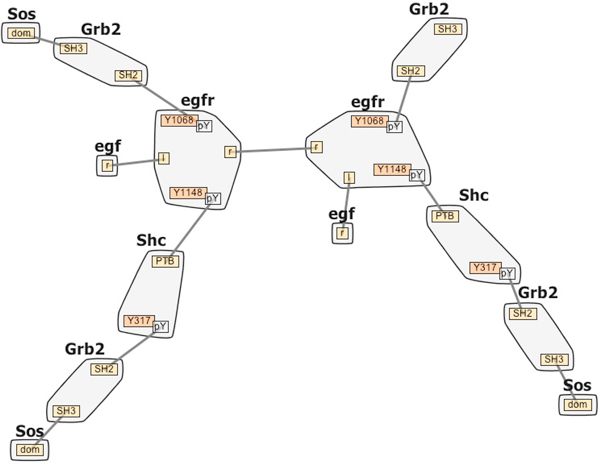
**The species graph**. The species graph is constructed similarly to the Contact Map. Shown is an example of a complex species containing thirteen molecules which is difficult to grasp from the text representation only.

The Simulation Results Viewer is linked to a Species Browser (Figure 7) in the Visualization Viewer in order to further help examine the resulting species. The Species Graph abstraction is constructed similarly to the Interactive Contact Map and alleviates the task of analyzing resulting species. Specifically, the full network generation of a model creates many new chemical species. When a CDAT file is opened in the Simulation Results Viewer, the list of all of these species is displayed below the results file tree viewer. Species from the list can be selected and visually represented in the Species Browser. Similarly, when viewing the list of observables associated with a specific GDAT file, nodes associated with an observable can be expanded to see all the species that contain the chemical species used to define that observable. Selecting any of these species will also cause them to be displayed in the Species Browser. Finally, right click context menus can be used to select text in the NET files and then to display the selected species.

### Linked-views for visual debugging

Based on our on-site workflow analysis, RuleBender was designed to assist in the 3 phases of Rule-Based Modeling (model, simulate, analyze results) using at most two panes at once: the Model Editor and the Visualization Viewer for model construction, or the Simulation Results Viewer plus the Visualization Viewer for results analysis. During modeling, the complementary views interact in order to assist with model exploration and debugging, whereas in the results analysis phase the Species Browser is used to visually show the chemical compounds that are being observed and the species that are created during network generation.

During the modeling phase the Model Editor is used in conjunction with the Visualization Pane. The user edits the text model in the Model Editor while the Visualization Pane displays the Contact Map and Influence Graph visual representations of the model. In addition to concurrent viewing of representations, interactions with the visualizations are propagated to the other views in order to visually link the model elements. Selections of model elements in the Contact Map, including compartments, molecules, domains, domain states, rules, and multiple rules (graph edges), result in Model Editor selections of the text that define the selected element (Figure 1). Simultaneously, the Contact Map selections of rules result in the selection of the nodes and associated edges that represent those rules in the Influence Graph. Similarly, selecting rule nodes in the Influence Graph causes Model Editor text highlighting of the rule text and the displaying of the bubble sets overlay that represents the rule in the contact map.

#### Detail view

While the visualization of the textual model helps with global knowledge of the system being created, the specific details of model elements are important during debugging and exploration. For this reason, the Detail Pane, shown in the upper right of Figure 1, displays relevant textual data in a table format for the currently selected visual element. The selection of any visual element displays the name, BNGL text definition for that element, and containing element where appropriate. The details table for molecule selection also shows a list of external links to online databases, such as Uniprot and Pathway Commons, which have more information about that element. Domain site selection also gives information about existing states, and state selection shows a list of rules that can affect the states. Rule information is also shown in the details view, such as the rule identifier and rates.

Linking the Model Editor, the Contact Map, the Influence Graph, the Species Browser, the Detail View, and the Results View assists the modelers in creating and debugging rule-based models. The multiple representations have complementary strengths in debugging model construction, as shown in our next section. Additionally, both the Contact Map and Influence Graph visualizations enable quick identification of orphan molecules or rules that do not interact with other molecules/rules, thus further supporting understanding and debugging of the models.

## Validation and results

Our next contribution is an evaluation of the utility and usability of RuleBender, with the following three components: (i) a demonstration of RuleBender's debugging capabilities on two case study models from our target user collaborators, who are systems biology researchers; (ii) a qualitative evaluation of the system at a biology research lab, gathered through surveys and interviews; and (iii) feedback from usage of the system as an educational tool.

### Case studies

#### EGFR

This model describes early events in biochemical signaling through the epidermal growth factor receptor (EGFR) which leads to differentiation and growth signals in cells. Dysregulation of signaling pathways activated by EGFR occurs in nearly all forms of cancer and mutations of EGFR and molecules activated downstream of EGFR are found in cancer cells at high frequency.

A senior systems biology researcher constructed an RBM model that is capable of predicting the dynamics of 356 molecular species, which are connected through 3749 unidirectional reactions. The researcher commented on the usefulness of the compact contact map visualization for showing what molecules can be connected in a complex, while still capturing the complexity of the system. He then noted that the visualizations highlighted the importance of the Shc aggregate (Figure 2) for recruitment: the key molecule Sos can be recruited to receptor in two different ways, through EGF-induced formation of EGFR-Grb2-Sos and EGFR-Shc-Grb2-Sos assemblies at the plasma membrane (note the corresponding paths in Figure 2). The highlighted rule also indicates that EGFR dimerization (formation of the compound through the joining of two molecules) is a necessary condition for this recruitment to take place. According to the researcher, these observations were tricky to see from the text-based representation, and easily missed without RuleBender. The researcher has adopted RuleBender as a research tool and is using it as their primary interface to RBM.

#### Lyn-Binding

**Figure 8:**
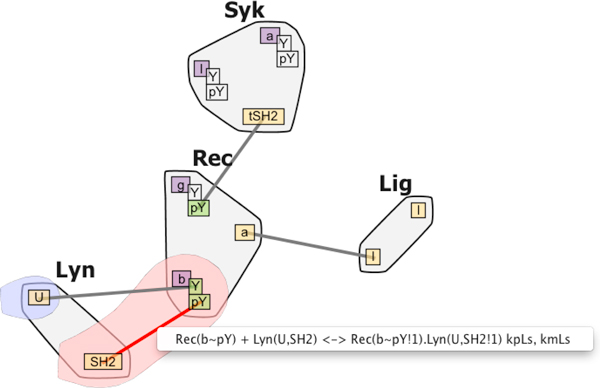
**Lyn-binding Contact Map**. Contact Map visualization for the Lyn-Binding model.

The Lyn-Binding represents early events in the antibody biochemical signaling process and is typically introduced as an exercise to junior researchers. The processes in the model are characteristic to allergic reactions, as well as to a system's response to injury or inflammation. RBM researchers have built a detailed mathematical model of reactions involving the receptor FcεRI (Rec), the enzyme Lyn, Syk, and a bivalent ligand (Lig) that aggregates Fc*ε*RI [35], all shown in Figure 8. The model makes it possible to test the consistency of mechanistic assumptions with data that alone provide limited mechanistic insight. The signaling network triggered by Fc*ε*RI plays a critical role in allergic responses and contains several targets for existing and proposed therapies for allergies.

In the model, signaling is initiated by the binding of ligand Lig to the receptor Rec, which leads to the formation of an aggregate containing two receptors. Lyn is recruited to these aggregates through binding to one of the receptors. There are two modes by which Lyn can associate with the receptor, one weak and one strong, depending on whether the receptor is already phosphorylated or not. Several novice researchers were given a partial model of this network, and asked to add the correct rule for the low-affinity binding of Lyn to the unphosphorylated *b *subunit via its *U *(unique) domain. To prevent a single Lyn molecule from bridging two separate receptors, they need to prevent the Lyn-receptor binding from occurring if the Lyn SH2 domain is already bound.

**Figure 9:**
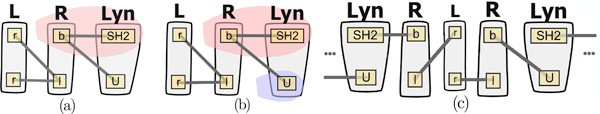
**Lyn-binding debugging**. Reduced view: Ligand notation shortened to L and Rec shortened to R. If the user programs the rule that binds Lyn to Rec incorrectly (see Table 2), the corresponding contact map in (a) is missing the rule context information. The correct binding leads instead to the visualization in (b); the presence of the blue bubble set alerted the researcher to the difference and allowed them to debug their RBM. The incorrect formulation would allow at run time for the creation of the infinitely binding chain shown in (c).

The researchers used RuleBender to debug their construction and simulation of this process. The contextual information, as well as the state information, turned out to be essential in constructing the Lyn-binding rule. Without making sure that the rules require that the other site be unbound, it would be possible for Lyn to bridge two separate receptors, thus potentially forming an infinitely binding chain (Figure 9a and 9c). This small error was not readily visible in the text-based model without careful review, and was thus a major source of frustration. Although the researchers routinely praise the benefits of RuleBender syntax highlighting, integrated execution of simulations and result viewing, in this instance they were only able to track down the error-source through the bubble-set reaction center and context visualization. Table 2 shows the correct and incorrect rule formulation, while Figure 9a and 9b show a reduced view of the resulting contact map for both the correct and incorrect formulation (no distinction evident). However, by using the bubble sets representation to explore the context and center of each reaction rule, the researchers noticed the missing context information in the incorrect rule formulation (highlighted with a blue bubble in the correct formulation).

**Table 2:** Lyn-Binding correct and incorrect rule formulation.

	Rule Text
Correct	*Rec*(*b*) + *Lyn*(*SH*2, **U**) *< - > Rec*(*b*!1)*.Lyn*(*SH*2!1, **U**)
	*Rec*(*b*) + *Lyn*(*U*, **SH2**) *< - > Rec*(*b*!1)*.Lyn*(**SH2**, *U*!1)
Incorrect	*Rec*(*b*) + *Lyn*(*SH*2) *< - > Rec*(*b*!1)*.Lyn*(*SH*2!1)
	*Rec*(*b*) + *Lyn*(*U*) *< - > Rec*(*b*!1)*.Lyn*(*U*!1)

The bold domains are omitted in the incorrect rules.

Junior researchers in the lab found the contact map and species browser visualizers *"most useful." *At the time, they commented that the influence graph had a nice look as well, but its main limitation were that the rules were difficult to track. The feedback led to several new iterations through the prototype, in particular, to the current influence graph visualization, in which nodes are labeled with rule mnemonics, as well as to the current design of linked views, where interacting with a graph node highlights the corresponding rule information in the text editor view.

### Qualitative evaluation

A series of interviews, as well as a pilot survey were conducted among four expert rule-based modelers from the Department of Computational Biology in order to evaluate the relative merits of the various RuleBender components. Three of the expert users had already adopted RuleBender as their primary tool for research, while the last one had used the system for less than one month. Based on our analysis of the tasks typically performed in RBM, as well as on our analysis of error sources, the users were asked to rate on a scale of 1 to 5 (much harder to much easier) the usefulness of RuleBender compared to command-line RBM with respect to the tasks listed in Table 1. The feedback shows that all the expert users found RuleBender significantly easier to use compared to BioNetGen command-line mode without visual interface, especially for tasks that require integration of the RBM workflow. The expert users were also asked to rate the relative usefulness of the various components of RuleBender, also on a scale of 1 to 5 (not helpful to essential). The visual representations and linked views were rated as useful, while syntax highlighting/checking, journaling of results, integrated execution of simulations, displaying the reaction center/context via bubble sets and interactive plotting in the result viewer were uniformly rated as very helpful or even essential. In particular, we note that adding the bubble sets capability increased the rating of the contact map from useful to very useful. In addition, the expert users highly recommended RuleBender as a teaching aid as opposed to BioNetGen in command-line mode.

Interview feedback remarked that RuleBender was easy to use, it was lightweight and cross-platform, and required minimal installation. Researchers commented that, based on their 10 year-long experience, tools lacking the above characteristics would just not be used. They also insisted on the benefits of a standalone system as opposed to a web-based application on account of latency; they explained that, unlike bioinformatics applications, systems modeling is typically CPU-bound.

### Educational use

RuleBender has been successfully deployed and used as a RBM educational tool in undergraduate/graduate classrooms at PITT, CMU, and Yale, as well as in a number of RBM workshops. Feedback from the instructors regarding the value of RuleBender was extremely positive (*"RBM without RuleBender was a no starter for the students"*, and *"The difference between teaching RBM without and with RuleBender is like the difference between night and day"*). RuleBender had *"a nice feel and interface"*, and was *"incredibly easy [...] to download and use"*. The system was *"definitely simpler than running simulations through the other [Matlab] interface, and could do just about everything we needed for the class assignments." *Finally, comments delivered the instructors' and students' excitement about RuleBender (*"a great start", "excited to see its future development!"*), as well as wish-lists for future features.

We note that in the 10 months following the open source release of RuleBender to the biology community, the system has been downloaded by 299 unique page visitors. The number of downloads comprises both research and educational use, however 94 downloads originate from outside of the United States and typically the temporal access patterns do not tightly coincide with classroom use.

## Discussion and conclusion

The user feedback (both at the expert and novice level) emphasizes that any tool that supports RBM must allow the user to build, simulate, and analyze models in an efficient workflow. We found that our visual framework efficiently creates such an RBM workflow by integrating model creation, simulation and analysis. As a measure of success, our users quickly adopted the tool as their main interface to RBM. Further feedback from the survey and interviews emphasizes that RuleBender is a user-friendly research and educational tool.

The results shown in the EGFR and Lyn-binding case studies demonstrate the benefits of visualization in exploring and explaining modeling errors. In these instances, RuleBender helped the researchers correctly and accurately gather observations and insights that were difficult to make otherwise.

The contact map visual representation helped the users see the model that they had written in a way that clarified its physical structures. Bubble sets made a major difference in how useful the users found this representation. The influence graph, in turn, was praised for its ability to identify orphan nodes and subsets of rules, and give insight into the signal firing process. The combined representations thus have complementary strengths. Although the local and global views of the models and their results are fragmented across multiple views, when combined in linked views and with details on demand, these views allowed the users to overcome several modeling pitfalls.

The contact map and influence graph representations were regarded as helpful additions to the tool, however, these visualizations may be further improved with biologically motivated or feature emphasizing layouts. In terms of scalability, models range in size from a few molecules and rules to dozens of molecules and hundreds of rules. Contact maps are reasonably scalable, but for large models the global influence graphs can become overwhelming despite zooming and drill-in capabilities. Furthermore, some biologists prefer symbolic forms to diagrammatic representations. Future work will focus on these areas with particular emphasis on scalability.

In terms of limitations, although our task analysis identified several types of errors in model construction, from the syntactic level to the biological level, RuleBender focuses primarily on detection of syntactic and semantic errors, with support for parameter scanning. Detection of biological errors is a far more difficult task, and may require the development of expert systems.

Furthermore, we note that RuleBender responds satisfactorily to all the tasks identified through our RBM task analysis, with the exception of *T*8 "Parameter estimation". Although journaling (keeping track of multiple simulations) and the species and results browsers are (according to the feedback) correct steps into alleviating this task, seamless integration with parameter estimation scripts appear to be important here and a direction of future work. A step further, and beyond the current scope of this work, is using the visual interface to create models, not only to debug them.

In terms of lessons learned from this collaboration, we found that a tight iterative prototyping loop was essential. The end users of RuleBender (both expert and novice) were also enthusiastic testers, and the cross-pollination of ideas is leading to further extensions of both the modeling language and the visual tools. Furthermore, we emphasize that essential traits of such tools include engineering characteristics such as cross-platform, stand-alone, and ease of installation. In introducing RuleBender to novice users, recording the steps taken to perform various designed exercises may be a valuable way to identify potential recurring user issues.

Rule-based modeling of systems arises in other domains outside of biology, for example state-machine specification, process calculi, or semantic-web applications. Solutions to scalability issues such as modularization or the development of typed systems transcend the specific domain boundaries, and are complementary to our visualization approach. We expect, however, that because of the complexity of biological networks (one complication here is that the network biochemistry of these systems does not have easily recognizable modular decompositions) effective visualization will be an integral element of rule-based modeling frameworks.

In conclusion, we introduced a novel, powerful tool for the development of RBMs. The tool makes RBM accessible to users with a wide range of computational experience, while providing a uniform interface across computing platforms. The support of RBM creation, debugging, and interactive visualization expedites the RBM learning process and reduces model construction time; while built-in model simulation and analysis with multiple linked views streamline the execution and analysis of newly created models and generated networks. A development cycle that includes close interaction with expert users allows RuleBender to better serve the needs of the systems biology community.

## List of abbreviations used

RBM: Rule-Based Model; BNG: BioNetGen; BNGL BioNetGen Language; EGFR: Epidermal Growth Factor Receptor.

## Competing interests

The authors declare that they have no competing interests.

## Authors' contributions

YS wrote the initial BNGL editor and contact map prototype. WX implemented the interactive influence graph, results analysis capabilities, and details view. AMS designed and implemented the user interface, and implemented the interactive contact map and linked views. JRF provided expert systems biology feedback and helped direct the design of the tool. GEM conceived of the project, designed user studies, and directed design and implementation of the tool. AMS, WX, JRF, and GEM contributed to the manuscript.
